# Expression and functional analysis of the Propamocarb-related gene *CsDIR16* in cucumbers

**DOI:** 10.1186/s12870-018-1236-2

**Published:** 2018-01-18

**Authors:** Chunhong Liu, Zhiwei Qin, Xiuyan Zhou, Ming Xin, Chunhua Wang, Dong Liu, Shengnan Li

**Affiliations:** 1 College of Horticulture and Landscape Architecture, Key Laboratory of Biology and Genetic Improvement of Horticultural Crops (Northeast Region), Northeast Agricultural University, Harbin, 150030 China; 2 Department of Applied Chemistry, College of Science, Northeast Agricultural University, Harbin, 150030 China

**Keywords:** Cucumber, Cucumber downy mildew, Propamocarb, Dirigent protein, *CsDIR*16

## Abstract

**Background:**

Cucumber downy mildew is among the most important diseases that can disrupt cucumber production. Propamocarb, also known as propyl-[3-(dimethylamino)propyl]carbamate (PM), is a systemic carbamate fungicide pesticide that is widely applied in agricultural production because of its high efficiency of pathogens control, especially cucumber downy mildew. However, residual PM can remain in cucumbers after the disease has been controlled. To explore the molecular mechanisms of PM retention, cucumber cultivars ‘D9320’ (with the highest residual PM content) and ‘D0351’ (lowest residual PM content) were studied. High-throughput tag-sequencing (Tag-Seq) results showed that the *CsDIR16* gene was related to PM residue, which was verified using transgenic technology.

**Results:**

We investigated the activity of a dirigent cucumber protein encoded by the *CsDIR16* in gene response to stress induced by PM treatment. Gene-expression levels of *CsDIR16* were up-regulated in the fruits, leaves, and stems of ‘D0351’ plants in response to PM treatment. However, in cultivar ‘D9320’, *CsDIR16* levels were down-regulated in the leaves and stems after PM treatment, with no statistically significant differences observed in the fruits. Induction by jasmonic acid, abscisic acid, polyethylene glycol 4000, NaCl, and *Corynespora cassiicola* Wei (Cor) resulted in *CsDIR16* up-regulation in ‘D0351’ and ‘D9320’. Expression after salicylic acid treatment was up-regulated in ‘D0351’, but was down-regulated in ‘D9320’. *CsDIR16* overexpression lowered PM residues, and these were more rapidly reduced in *CsDIR16*(+) transgenic ‘D9320’ plants than in wild-type ‘D9320’ and *CsDIR16*(−) transgenic plants.

**Conclusions:**

Analyses of the *CsDIR16*-expression patterns in the cucumber cultivars with the highest and lowest levels of PM residue, and transgenic validation indicated that *CsDIR16* plays a positive role in reducing PM residues. The findings of this study help understand the regulatory mechanisms occurring in response to PM stress in cucumbers and in establishing the genetic basis for developing low-pesticide residue cucumber cultivars.

**Electronic supplementary material:**

The online version of this article (10.1186/s12870-018-1236-2) contains supplementary material, which is available to authorized users.

## Background

Pesticides are among the most widely used chemicals in the world. With their application in modern agriculture, up to 80% of crop yield were protected from pest and weeds [1]. However, contamination of products and environment was triggered by the extensive use of pesticides in most regions [2]. Food consumption is one of the most common routes of pesticide exposure in consumers [3]. Pesticide residues represent a major food safety issue since some of them are suspected to mutagenic, carcinogenic, and teratogenic activities. For example, many pesticides cause acute toxicity, as well as sublethal effects by causing the endocrine disorders, sperm quality decline and reproductive development abnormalities [4–7]. Considerable efforts have been made to reduce the pesticide residues of agricultural products through both traditionally breeding programs and contemporarily genetic transformation. Identifying biotransformation mechanisms of pesticide residues at the molecular level has emerged as a new approach for studying pesticide residues [8, 9].

Downy mildew is a devastating disease affecting cucurbits that is caused by infection by zoospores. The associated lesions can induce chlorosis or yellowing, and further destroy the entire leaf in a few days [10]. Propamocarb, also known as propyl-[3-(dimethylamino) propyl] carbamate (PM), is a low-toxicity fungicide with systemic activity after absorption through leaves, stems, and roots, and by transportation throughout treated plants via the vascular system [11]. The fungicide PM has good protective and curative activities against downy mildew with no phytotoxicity effects in fruits and vegetables, such as tomatoes, potatoes and cucumbers [12–16]. PM causes slight cytotoxicity to cortical neurons and moderately effects on the intracellular membrane potential, glucose consumption, ATP levels, and the cytoskeleton [17]. Bone-marrow micronucleus and chromosome aberration test results with Swiss albino mice suggested that PM was not genotoxic in mouse bone marrow in vivo, but had cytotoxic effects [18]. These data indicate that PM residues in fruits and vegetables pose potential health risks in humans.

Cucumber downy mildew, caused by *Pseudoperonospora cubensis* (Berk. & M.A.Curtis, Rostovzev)*,* is an important leaf disease that can spread quickly and reduce cucumber yields [19, 20]. Both production and quality of cucumber have thus been affected, and then led to economic losses. PM is an effective control for downy mildew; however, PM residues can remain in cucumber plants after the disease has been controlled. PM residues may accumulate at levels higher than the international maximum residue limits (MRLs) [21]. Improving the yield and quality of cucumber products, ensuring food safety for consumers, and improving the international competitiveness of Chinese vegetable products are important objectives.

Despite mounting concerns regarding pesticide residues on vegetables, numerous scientific advances have been made in detection technologies and physiological mechanisms influencing pesticide-residue levels. Methods for assessing PM-residue levels have been established [22]. Twenty-eight cucumber germplasm resources have been collected and used to compare PM residues among cultivars. Among them, cultivar ‘D0351’ was found to have the lowest PM-residue levels, as well as ‘D9320’ the highest [21, 23]. To investigate the molecular mechanisms that determine PM-residue levels in cucumbers, high-throughput tag-sequencing (Tag-Seq) has been performed using Illumina analysis (based on the Solexa Genome Analyzer platform), in order to study gene-expression profiles in control and PM-treated fruit from ‘D0351’ plants [21]. Data from several studies have shown that pesticide residue levels are controlled by multiple genes [22]. Transcriptomic analysis revealed that *CsABC19* and *CsWRKY30* are 2 positive regulators of plant tolerance to PM stress. Overexpression of CsABC19 and CsWRKY30 in Arabidopsis enhanced tolerance to PM stresse and decreased PM residues [24, 25]. *CsDIR16* is another key gene involved in responses to PM stress, as revealed by transcriptomic analysis.

Dirigent proteins (DIRs) were first discovered by Davin et al. [26] in *Forsythia intermedia*. Subsequently, DIR and DIR-like family proteins have been reported in many plant species, including gymnosperms and angiosperms. The DIR gene family encodes several proteins involved in secondary metabolism, lignan and lignin formation biosynthesis [27–29], or responses to pathogen infection and abiotic stress [30–34]. DIRs lack a catalytically active (oxidative) center and function only as guiding proteins [35]. The mechanism of action is thought to involve capture of free radicals produced by the oxidation of coniferyl alcohol, and the FiDIR protein catalyze 8–8′ coupling to produce (+)-pinoresinol, the AtDIR5 and AtDIR6 proteins catalyze 8–8′ coupling to produce (−)-pinoresinol [36–38]. This is followed by intramolecular cyclization to increase total lignin accumulation and improve resistance to stress [39]. DIRs affect the acidity of lignin by changing the composition and connection of the lignin monomer, thereby reducing cellular damage caused by drought or water stress to help plants increase stress resistance [40]. The *DIR* genes serve a universal function in terms of stress resistance, but their roles in response to PM treatment have not been determined. The *CsDIR16* gene was upregulated in ‘D0351’ plants exposed to PM stress, which suggests that *CsDIR16* plays a vital role in plant responses to PM stress and may be of value for future production of transgenic cucumbers with enhanced PM stress responses.

## Results

### Cloning and bioinformatics analysis of *CsDIR16*

**Fig. 1:**
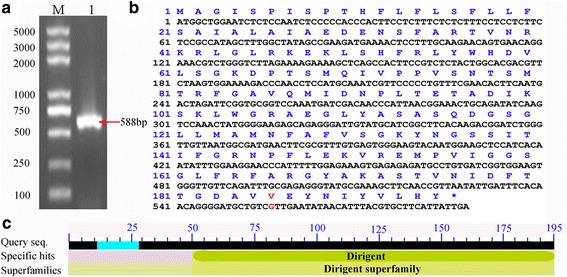
PCR-amplification and characteristics of the full-length coding region of *CsDIR16* cDNA from cucumber fruit of ‘D0351’. The domain of CsDIR16 protein sequence was determined by analysis with the Conserved Domain Database (CDD) of NCBI. **a** Agarose gel electrophoresis of PCR-amplified CsDIR16. Lane M: 5000-bp DNA Marker (Toyobo, Japan); Lane 1: PCR amplification of *CsDIR16*. **b** Nucleotide sequence and deduced amino acid sequence of *CsDIR16*. The initiation codon (ATG) and the terminator codon are marked with “*.” **c** Conserved domain prediction of the CsDIR16 protein sequence. Searching with the NCBI CDD showed that CsDIR16 belongs to the dirigent family and the dirigent superfamily

In this study, we investigated a cucumber PM-responsive gene (Csa4M280630.1), designated here as *CsDIR16*. The full-length cDNA sequences of *CsDIR16* were cloned from cucumber fruits of ‘D0351’ and ‘D9320’ by reverse transcription-PCR (RT-PCR) (Fig. 1a). Then the sequence of *CsDIR16* was confirmed by repeated sequencing. Comparison with the cucumber genome database (http://www.icugi.org/), the sequences of *CsDIR16* cloned from cucumber fruits ‘D9320’ showed no difference. However, a A-to-G mutation at position 556 was found in the sequences of *CsDIR16* cloned from cucumber fruits ‘D0351’, resulting in an I-to-V amino acid change at position 186. *CsDIR16* has a 588-base pair (bp) open-reading frame (ORF) encoding 195 amino acids, and the calculated molecular weight is 21.55 kDa (Fig. 1b).

**Fig. 2:**
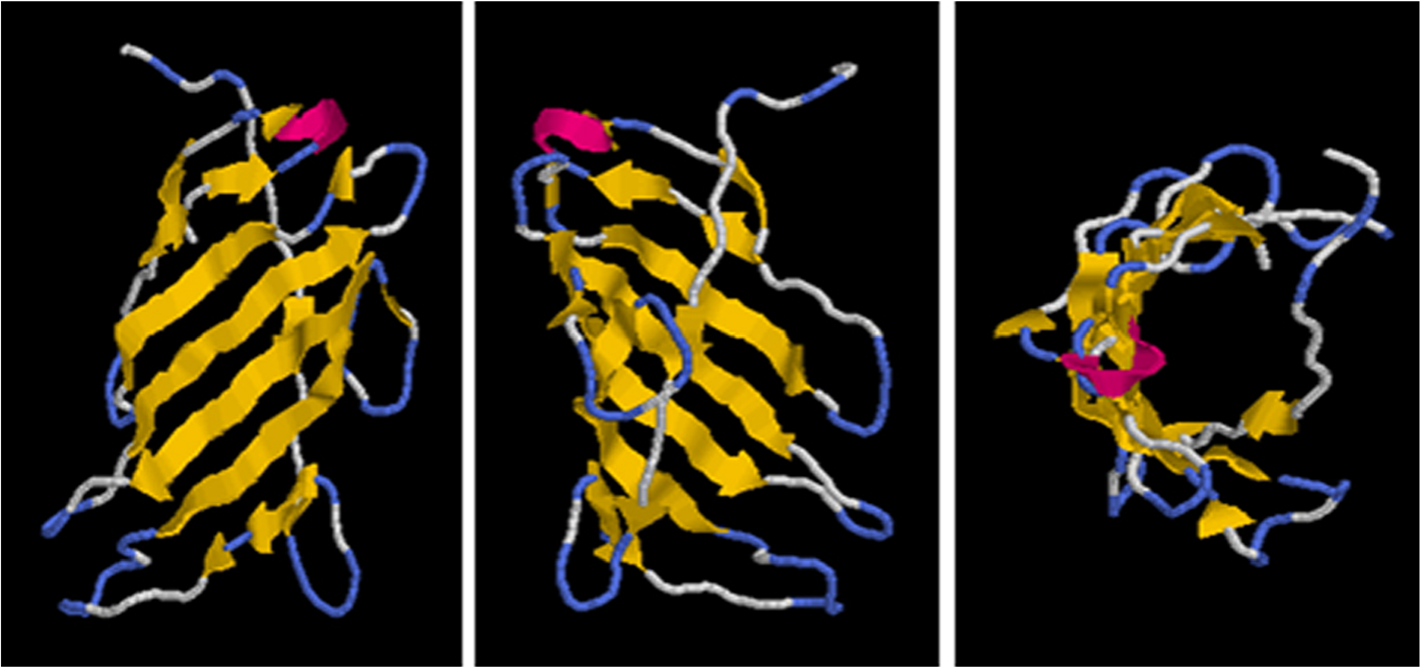
Three-dimensional structures of the CsDIR16 protein from different views. β-folds and β-turns are marked in yellow and pink, respectively

The CsDIR16 protein contains a dirigent protein domain (PF03018) (Fig. 1c) and 5 conserved motifs (Additional file 1: Figure S1) [40]. An N-terminal signal peptide and a cleavage site between amino acids 28 and 29 were predicted in the protein by SignalP 4.0 (Additional file 1: Figure S2). A transmembrane helix between residues 10 and 27 was predicted by TMHMM 2.0 (Additional file 1: Figure S3). CsDIR16 has 2 N-glycosylation sites at amino acids 77 (Asn) and 135 (Asn). Such N-glycosylation sites have been found in BhDIR and can be regarded as a feature of secreted proteins [31]. Figure 2 showed the predicted 3-dimensional structures of the CsDIR16 protein. Structures of the CsDIR16 protein were modeled using CPHmodels 3.2 to generate atomic coordinates, using Rasmol windows software output CsDIR16 to visualize the 3-dimensional protein structure. Analysis of the predicted 3-dimensional structure showed that CsDIR16 has a 14 β-fold (yellow) and 1 β-turn (pink) that constitute a typical β-barrel structure for a DIR protein, with a hydrophobic chamber in the center [36, 37]. The I-to-V mutation mentioned above involves two alternative hydrophobic amino acids with similar structures, which were not located in the β-fold. The 3-dimensional structure showed that the mutation was not predicted to critically affect the construction of the β-barrel.

Cis-acting elements of the *CsDIR16* promoter region were predicted using PlantCARE online analysis tools. Common cis-acting elements such as TATA and CAAT boxes; light-responsive elements such as Box4 and Box I. Cis-acting elements specifically linked to stress responses such as P-box (gibberellin-responsive element), TC-rich repeats (involved in defense and stress responsiveness), the TCA element (a cis-acting element involved in salicylic acid responsiveness), the TGA element (an auxin-responsive element), and a W box (WRKY-binding site) were identified in the promoter region (Additional file 1: Table S1). The presence of these stress-related cis elements showed that the promoter region of *CsDIR16* responded to various kinds of stress signals and that *CsDIR16* expression was regulated by several stress factors.

### Phylogenetic tree of CsDIR16

**Fig. 3:**
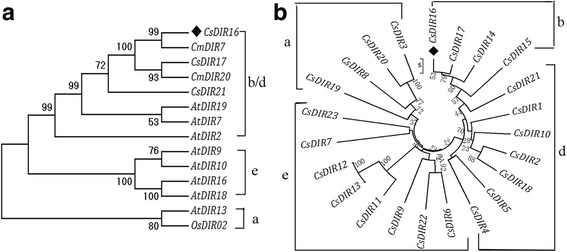
Phylogenetic analysis and sequence alignment of CsDIR16*.* **a** Phylogenetic relationships of CsDIR16 with homologous proteins from other plant species. **b** Phylogenetic tree of all CsDIRs. The homologous sequences were from species showing the highest *CsDIR16* sequence similarities. The amino sequences were subjected to phylogenetic analysis using the neighbor-joining method in MEGA7.0.20 software, with 1000 bootstrap replicates. The GenBank accession numbers are as follows: AtDIR2 (NP_199065.1), AtDIR7 (NP_187974.1), AtDIR9 (NP_181475.1), AtDIR10 (Q9SIA8.1), AtDIR13 (NP_192858.1), AtDIR16 (NP_189044.1), AtDIR18 (NP_193094.1), AtDIR19 (NP_176113.1), CmDIR7 (XP_008449790.1), CmDIR20 (XP_008449789.1), OsDIR02 (BAS72036.1), CsDIR1 (KGN63770.1), CsDIR2 (XP_004138340.1), CsDIR3 (KGN64478.1), CsDIR4 (XP_004147025.1), CsDIR5 (XP_004147026.1), CsDIR6 (XP_011649995.1), CsDIR7 (KGN55603.1), CsDIR8 (XP_004134021.1), CsDIR9 (XP_004134245.1), CsDIR10 (XP_004153955.1), CsDIR11 (XP_004146539.1), CsDIR12 (XP_004146540.1), CsDIR13 (XP_004146540.1), CsDIR14 (KGN54084.1), CsDIR15 (KGN54085.1), CsDIR16 (XP_004142157.2), CsDIR17 (XP_004142156.1), CsDIR18 (XP_011653564.1), CsDIR19 (KGN50509.1), CsDIR20 (XP_004147710.2), CsDIR21 (XP_004140684.2), CsDIR22 (XP_004144592.1), CsDIR23 (XP_004139895.1)

A phylogenetic tree of CsDIR16 and other related dirigent proteins in other plant species was constructed using Mega7.0 software (Fig. 3a). This analysis showed that CsDIR16 belongs to the DIR-b/d subfamily; the phylogenetic tree revealed that CsDIR16 has high homology to other dirigent-like proteins and shares 99% similarity with the *Cucumis melo* protein CmDIR7. CsDIR16 and Arabidopsis AtDIR proteins are very divergent in their evolutionary relationship. Several studies revealed that different subfamilies with different functions. DIR-a subfamily genes encode proteins involved in lignan and lignin formation biosynthesis [27–29], and DIR-b/d subfamily genes respond to pathogen infection and abiotic stress [30–34].

Twenty-three transcripts were identified in the cucumber genome sequence as possible members of the dirigent family and were named CsDIR1 to CsDIR23, based on their order in the cucumber genomic sequence (Additional file 1: Table S2). A phylogenetic tree of CsDIRs was constructed using Mega7.0 software (Fig. 3b). This analysis indicated that these proteins belong to 4 subfamilies (DIR-a, DIR-b, DIR-d, and DIR-e). Transcriptome analysis [23] showed that only 4 (*CsDIR5*, *CsDIR7*, *CsDIR10*, *CsDIR16*) of the 23 *CsDIR* genes in cucumber responded to PM treatment (Additional file 1: Table S3). Of these 4 genes, *CsDIR16* had the highest expression level.

### Subcellular localization of the CsDIR16 protein

**Fig. 4:**
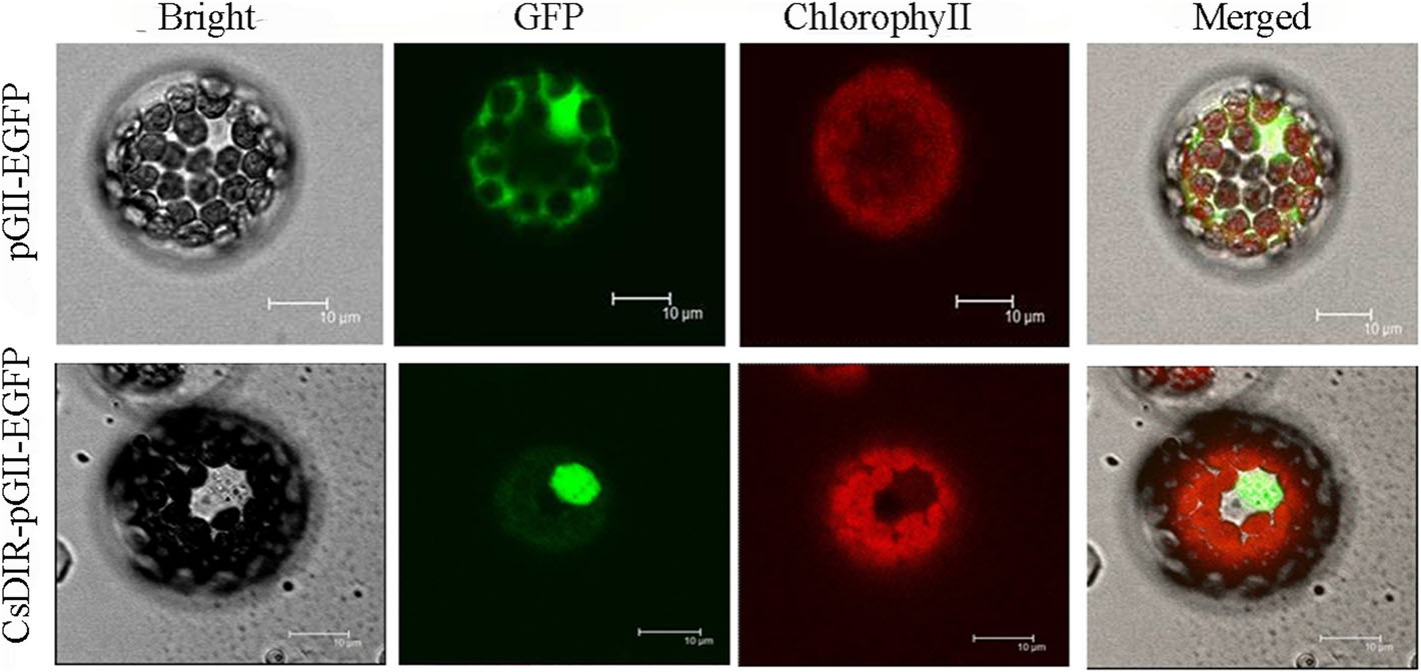
Subcellular localization of the *CsDIR16*-pGII-EGFP fusion protein in *Arabidopsis* protoplasts. Images show protoplasts prepared from 3- to 4-week-old Arabidopsis leaves expressing *CsDIR16*-pGII-EGFP (upper row) or pGII-EGFP (bottom row). Bright-field illumination, GFP fluorescence, chlorophyll fluorescence, and an overlay of GFP and chlorophyll fluorescence are shown. Scale bars, 10 μm

The subcellular localization of the CsDIR16 protein was investigated using a *CsDIR16-eGFP* fusion gene driven by a 35S promoter; transient expression in Arabidopsis protoplast cells showed that the CsDIR16-eGFP fusion protein was enriched in the nucleus (Fig. 4). The results clearly indicated that CsDIR16 is a nuclear-localized protein.

### Expression pattern of *CsDIR16* in response to PM treatment

**Fig. 5:**
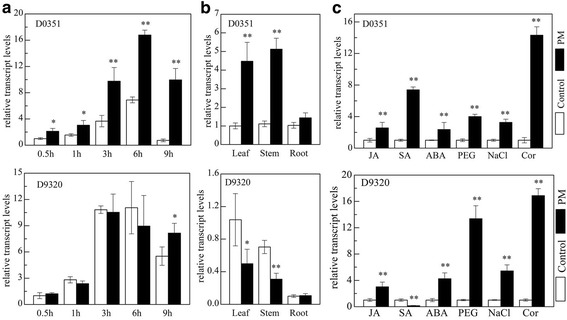
Expression pattern of *CsDIR16* in cucumber ‘D0351’ and ‘D9320’ after PM treatment and different stresses. **a** Relative transcript levels in fruits at 0.5, 1, 3, 6, and 9 h after PM treatment. **b** Relative transcript levels in leaves, stems, and roots at 6 h after PM treatment. **c** Relative transcript levels in 3-leaf seedlings after treatment with JA, SA, ABA, PEG4000, NaCl, or Cor. Controls were treated with distilled water. *EF1α* was detected as the internal control gene. Three biological replicates per treatment and three technical replicates were analyzed per sample. Significant differences are indicated by asterisks (**P < 0.01, *P < 0.05 by Student’s *t*-test), compared with the corresponding control. Error bars indicate the standard deviation of the mean

The expression patterns of *CsDIR16* in the fruit of cultivars ‘D0351’ and ‘D9320’ were determined in control and PM-treated plants (Fig. 5a). In the ‘D0351’ cultivar, the expression level of *CsDIR16* gene significantly increased at 3 h post-PM treatment, plateaued at 6 h, and then gradually decreased over time*.* The relative fold-changes of expression were 2.13, 1.97, 2.66, 2.43, and 13.77-fold at 0.5, 1, 3, 6, and 9 h after treatment, respectively. In contrast, *CsDIR16* expression followed a different pattern in the ‘D9320’ cultivar. Peak *CsDIR16* expression was seen at 3 h post-treatment, and no statistically significant differences were observed compared to control plants except at 9 h. Differences in *CsDIR16* gene expression in response to PM were observed between the lowest PM-residue cultivar ‘D0351’ and the highest PM-residue cultivar ‘D9320’, indicating that the *CsDIR16* gene might be closely associated with PM metabolism. *CsDIR16* expression was induced by PM treatment in the fruit of ‘D0351’, suggesting that this gene serves a crucial role in metabolizing PM. However, no significant up-regulation of *CsDIR16* was found in ‘D9320’, indicating that *CsDIR16* is not the main metabolically related gene for PM residues in this cultivar.

To compare *CsDIR16-*expression patterns in response to PM in different tissues, we analyzed leaves, stems, and roots at 6 h post-treatment in ‘D0351’ and ‘D9320’ plants (Fig. 5b). *CsDIR16* was expressed in all 3 tissues, but the expression patterns differed between the 2 cucumber cultivars. *CsDIR16* was upregulated by PM treatment in the leaves and stems of ‘D0351’. In contrast, *CsDIR16* was expressed at extremely low levels in the leaves and stems of ‘D9320’. No statistically significant differences were observed in the roots of ‘D0351’ and ‘D9320’. These results indicated that *CsDIR16* expression was significantly induced by PM treatment in the fruit, leaves, and stems of ‘D0351’, suggesting that these are the major organs for metabolism of the fungicide.

### Expression pattern of *CsDIR16* in response to hormone induction and various exogenous stresses

*DIR* genes are involved in various hormone responses and exogenous stresses [30–34]. In this research, the effects of other potential stressors on *CsDIR16* expression were examined. The lowest PM-residue cultivar ‘D0351’ and the highest PM-residue cultivar ‘D9320’ were treated with jasmonic acid (JA), salicylic acid (SA), abscisic acid (ABA), polyethylene glycol 4000 (PEG4000), NaCl, or Cor. *CsDIR16* expression in ‘D0351’ was significantly up-regulated by treatment with each compound, with expression levels 2.57, 7.40, 2.37, 4.00, 3.27, and 14.32-fold higher than those in the control (Fig. 5c), respectively. *CsDIR16* expression in ‘D9320’ was significantly up-regulated by each compound, except for SA, and the relative fold-changes of expression were 3.02, 0.13, 4.24, 13.36, 5.44, and 16.88-fold higher, respectively, than that in the control (Fig. 5c). These results revealed that *CsDIR16* could be up-regulated by JA and ABA, indicating that *CsDIR16* may be involved in JA- and ABA-associated signaling pathways. The expression pattern after SA treatment was significantly higher in ‘D0351’ compared to ‘D9320’, reflecting the same pattern observed after PM treatment. These results indicated that *CsDIR16* expression was significantly induced by JA, SA, ABA, PEG4000, NaCl, and Cor stress, suggesting that *CsDIR16* might be involved in cultivar responses to various biological and abiotic stresses.

### *CsDIR16* overexpression enhanced PM metabolism in transgenic cucumbers

**Fig. 6:**
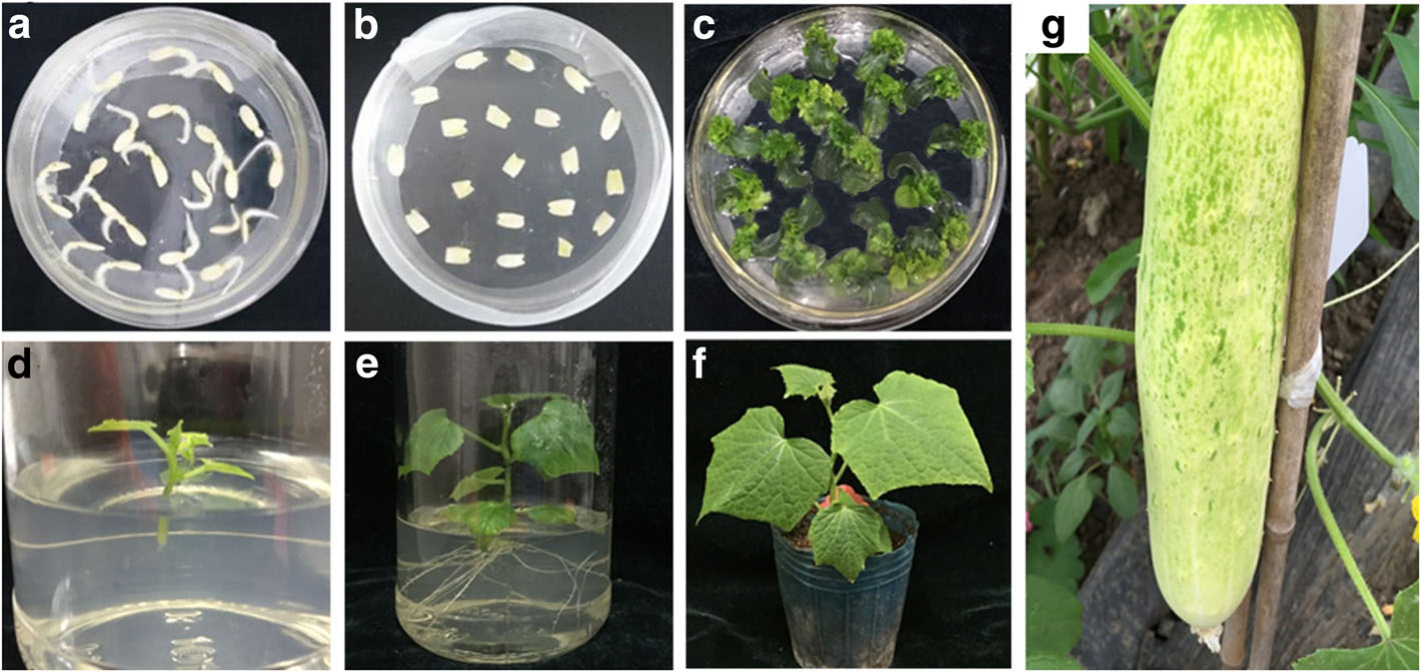
pCXSN-*CsDIR16*(±) genetic transformation of cucumber ‘D9320’. **a** Seed germination, **b** co-culture, **c** screening culture, **d** plant regeneration, **e** resistant seedlings taking root, **f** regeneration of resistant seedlings, **g** seed of transgenic plants

**Fig. 7:**
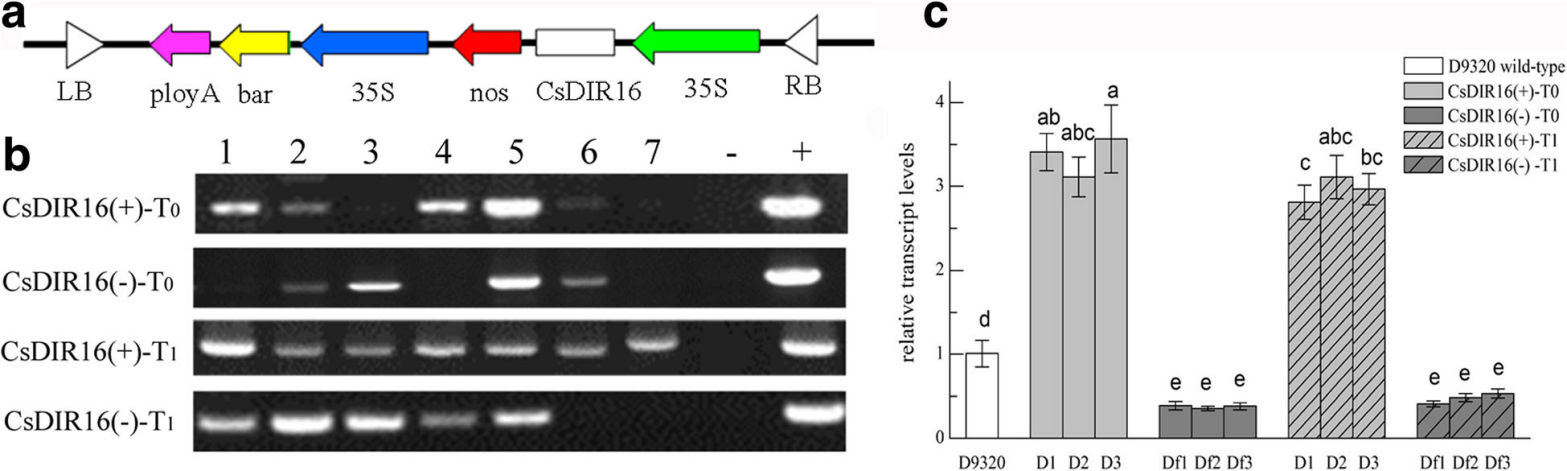
Characterization of *CsDIR16-*overexpression transgenic plants. **a** Schematic representation of the *CsDIR16*-overexpression plasmid. **b** Transgenic plants T_0_ and T_1_ were identified by PCR. **c** Relative *CsDIR16* transcript levels in transgenic plants T_0_ and T_1_. Error bars indicate the standard deviation of the mean. Superscripted letters represent significant differences at the 0.05 level, based on Tukey’s test

The over-expression vectors *CsDIR16*(+)-PCXSN and *CsDIR16*(−)-PCXSN were successfully transferred into the highest PM-residue cultivar D9320 using cucumber genetic-transformation technology (Fig. 6). We generated transgenic cucumbers overexpressing *CsDIR16* under the control of the strong constitutive CaMV35S promoter (Fig. 7a). Transgenic cucumbers T_0_ and T_1_ were identified by PCR (Fig. 7b), and the expression of *CsDIR16* was analyzed by quantitative real-time PCR (qRT-PCR; Fig. 7c). Both T_0_ and T_1_ transgenic cucumbers showed increased levels of *CsDIR16* expression (approximately 3-fold higher). In T_0_ and T_1_ *CsDIR16*(−) transgenic cucumbers, the level of expression was approximately 0.4 that of the normal control. Overall, we obtained 13 *CsDIR16*(+) and 15 *CsDIR16*(−) T_0_ transgenic cucumbers.

**Fig. 8:**
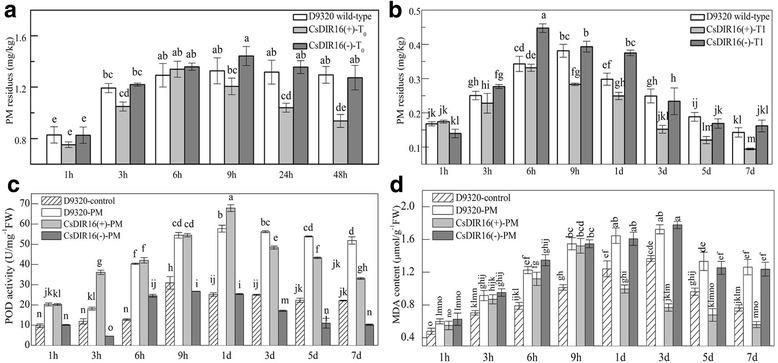
Quantitative analysis of propamocarb (PM) residues, peroxidase (POD) activity, and the malondialdehyde (MDA) content in transgenic plants. **a** Quantitative analysis of PM residues in T_0_ transgenic plants using gas chromatography. **b** Quantitative analysis of PM residues in T_1_ transgenic plants using gas chromatography. **c** Quantitative analysis of POD activity in T_1_ transgenic plants. **d** Quantitative analysis of the MDA content in the T_1_ transgenic plants. The means ± SD of 3 replicates are shown. Superscripted letters represent significant differences at the 0.05 level, based on Tukey’s test

We compared the levels of PM residues in wild-type ‘D9320’, and T_0_ and T_1_ *CsDIR16*(+) and *CsDIR16*(−) transgenic cucumbers. The level of residual PM was lower in *CsDIR16*(+) transgenic plants than in wild-type ‘D9320’ and *CsDIR16*(−) plants after PM treatment (Fig. 8a and b). The PM levels did not differ significantly between wild-type ‘D9320’ and *CsDIR16*(−) transgenic plants. As *CsDIR16* was not a main gene responsible for metabolic removal of PM in ‘D9320’, the presence of the antisense expression vector *CsDIR16*(−) did not affect PM metabolism in *CsDIR16*(−) transgenic cucumbers. Our results indicated that *CsDIR16* overexpression is likely to reduce PM residue levels by accelerating degradation of the fungicide.

### *CsDIR16* may require peroxidase (POD) activities to enhance PM metabolism in cucumbers

Pesticide molecules, just like toxic xenobiotics, could affect relevant enzyme activities and transform gene-expression patterns. Abiotic stresses in plants can cause excessive accumulation of reactive oxygen species (ROS). ROS are highly reactive and toxic that may lead to damage to proteins, lipids, carbohydrates, and DNA [31, 41]. Plants have defense mechanisms to protect against oxidative stress damage. POD is an antioxidant enzyme that scavenges ROS. Here, we measured POD activities after PM treatment in wild-type ‘D9320’, *CsDIR16*(+), and *CsDIR16*(−) T_1_ transgenic plants (Fig. 8c). After PM treatment, POD activity increased and peaked at 1 d in wild-type ‘D9320’ and *CsDIR16*(+) T_1_ transgenic plants. The *CsDIR16*(+) T_1_ transgenic cucumbers showed significantly more rapid reduction of POD activity compared with that in wild-type plants. *CsDIR16*(−) T_1_ transgenic line showed a similar trend to wild-type plants treated with water.

Malondialdehyde (MDA) is commonly used as a marker of oxidative lipid injury, whose concentration varies in response to biotic and abiotic stresses [42]. The MDA contents in wild-type ‘D9320’, *CsDIR16*(+), and *CsDIR16*(−) T_1_ transgenic cucumbers after PM treatment were determined (Fig. 8d). We found that MDA contents increased significantly and reached a peak at 9 h in the *CsDIR16*(+) T_1_ transgenic line, but this peak was only reached at 3 d after PM treatment in the wild-type ‘D9320’ cultivar and the *CsDIR16*(−) T_1_ transgenic line. After 9 h, the MDA content in *CsDIR16*(+) T_1_ transgenic plants decreased more rapidly compared with wild-type plants and *CsDIR16*(−) T_1_ transgenic plants. These results suggested that *CsDIR16* overexpression reduced PM residues through accelerating PM degradation.

## Discussion

Many plant genes are induced by biological and abiotic stresses, such as insects, fungi, drought, and high-salinity. Not only may these genes function in stress responses, but in stress tolerance as well [43, 44]. Gene-expression patterns are often associated with gene function [24]. In a previous study, the transcriptome changes in cucumber fruit of cultivar ‘D0351’ in response to PM treatment were analyzed by our research group. [21]. The transcriptome data indicated that *CsDIR16* showed the highest differential expression between plants treated with PM versus water. After treatment with PM, we found that only *CsDIR16* showed significantly differential expression between ‘D0351’ and ‘D9320’ (Fig. 5a and b). *CsDIR16* was significantly up-regulated in the fruits, leaves, and stems of the lowest-residue cultivar ‘D0351’. In the highest-residue cultivar ‘D9320’, *CsDIR16* was expressed at extremely low levels in leaves and stems, with no significant up-regulation in fruits. The expression pattern of *CsDIR16* was related to plant PM residues, which indicated that *CsDIR16* plays an important role in the response to PM.

To investigate the role of *CsDIR16* in PM responses, the *CsDIR16* gene was transformed into the wild-type ‘D9320’ cultivar (Fig. 4a–d) and the effects of *CsDIR16* overexpression on PM residues were measured. *CsDIR16*(+)-overexpressing transgenic cucumber plants showed lower PM residues and more rapid PM-residue reduction compared with wild-type and *CsDIR16*(−) plants (Fig. 8a and b). Dirigent proteins lack a catalytically active (oxidative) center and depend oxidases to biosynthesize lignin and lignan [35]. The 3-dimensional structure of CsDIR16 showed that it has a typical β-barrel structure with a hydrophobic chamber in the center (Fig. 2) and lacks an oxidative center.

POD is the key enzyme in the phenylpropanoid pathway. This pathway is significantly associated with the cucumber metabolism of PM [21]. POD activities increased in cultivar ‘D0351’ after treatment with PM, while lignin increased by 41.1% [45]. Here, we found that POD activity in *CsDIR16*(+) T_1_ transgenic plants fell more rapidly compared with that in wild-type plants, indicating that *CsDIR16* could increase POD-activity responses to PM. The reduction in POD activity in *CsDIR16*(+) T_1_ transgenic plants followed the rapid reduction in PM residues. We found that the MDA content increased in wild-type ‘D9320’ after PM treatment, suggesting that the fungicide may cause damage to the plants. The MDA content fell more rapidly in *CsDIR16*(+) T_1_ transgenic plants compared with wild-type plants, indicating that the *CsDIR16* gene can repair the plasma membrane and lead to a more rapid lowering of the MDA content. Based on these results, we speculate that PM activates the phenylpropanoid pathway, causing increased POD activity. According to this model, the phenoxy radicals scavenged by POD are oxidized by the CsDIR16 protein in the fruit, leaves, and stems to produce pinoresinol, which rapidly reduces the damage caused by PM by effectively diminishing the PM residues in ‘D0351’.

Phylogenetic analysis showed that CsDIR16 belongs to the DIR-b/d subfamily (Fig. 2a), which is responsive to abiotic stress. In our study, *CsDIR16* was significantly up-regulated after JA, SA, ABA, PEG4000, NaCl, and Cor treatments (Fig. 5c), showing that it influences responses to such stressors [31, 40, 46, 47]. The phytohormone SA acts as a signaling molecule and can induce plant stress responses under adverse conditions. The similarity in the expression patterns of *CsDI*R16 (Fig. 5b and c) after SA or PM treatments in leaves of ‘D0351’ and ‘D9320’ may indicate that the regulatory mechanisms induced by SA are different in ‘D0351’ and ‘D9320’, resulting in different levels of PM residues.

## Conclusion

In summary, the CsDIR16 protein localized to the cell nucleus and could respond to abiotic and biotic stresses. *CsDIR16* adapted to PM treatment and reduce the levels of residues in *CsDIR16*(+) transgenic plants through enhancing plant metabolism and physiological functions. Further research on *CsDIR16* function will provide additional genetic resources for breeding stress-resistant plants and may provide further insights into pesticide-stress mechanisms.

## Methods

### Plant materials and stress treatments

D0351 and D9320 used in this study were homozygous cucumber lines. The low-PM-residue cultivar ‘D0351’ and the high-PM-residue cultivar ‘D9320’ had been identified by Fangfang Liu [22]. The seeds were provided by cucumber research group of Northeast Agricultural University, Harbin, China. Seeds of ‘D0351’ and ‘D9320’ were germinated, and the seedlings were grown under following conditions: 25–30 °C day, 15–18 °C night; 60–75% relative humidity in a greenhouse at the College of Horticulture, Northeast Agricultural University, Harbin, China.

Young plants at 34 days after transplanting at the 3-leaf stage were used for expression analysis of *CsDIR16* in response to PM treatment. 8 mM PM solution was sprayed to similar sizes plants for 1 min until the surface of the leaves and fruits began to drip [21, 23, 24]. The leaves, stems, roots, and fruit peels (ca. 2 mm thick, 1 cm^2^, from the nodes of 10 fruit per plant, respectively) were sampled at 0.5, 1, 3, 6, and 9 h after treatment. Control plants were sprayed with distilled water.

Three-leaf seedlings were used to determine changes in *CsDIR16* expression after treatment with JA, SA, ABA, PEG4000, NaCl, and Cor. The plants were sprayed with 100 μmol/L JA, 100 μmol/L SA, or 100 μmol/L ABA [48]; leaves were harvested 12 h after treatment. Seedlings were irrigated with 50 mL 40% PEG4000; leaves were harvested 8 days after treatment. Seedlings were irrigated with 50 mL 400 mmol/L NaCl, once every 3 days; leaves were harvested 8 days after treatment. Seedlings were sprayed with 1 × 10^5^ colony-forming units/mL Cor; leaves were harvested 24 h after treatment. All samples were immediately frozen in liquid nitrogen and stored at − 80 °C until used for RNA extraction.

To measure the effects of *CsDIR16*(+) and *CsDIR16*(−) overexpression on PM residues, T_0_ transgenic cucumber young plants with similar levels of *CsDIR16* expression were sprayed with 8 mM PM solution. Leaves were harvested at 0.5, 1, 3, 6, and 9 h after treatment. Wild-type ‘D9320’ plants were sprayed as controls. Seeds of the T_1_ generation were obtained via the self-cross of the T_0_ generation lines. Three-leaf T_1_ transgenic cucumber seedlings with similar levels of *CsDIR16* expression were sprayed with 1 mM PM solution. Leaves were harvested at 1 h, 3 h, 6 h, 9 h, 1 d, 3 d, 5 d, and 7 d after treatment. Wild-type ‘D9320’ plants were sprayed as controls.

### Gene cloning and bioinformatics analysis of *CsDIR16*

Total RNAs (from leaves, stems, roots, and fruit peels) were extracted using the TRIzol reagent (Invitrogen). Total RNA (1 μg) was reverse transcribed with a ReverTra Ace qPCR RT Kit (Toyobo, Japan) for cDNA synthesis. The cucumber genome database was applied for searching the full-length coding sequences (CDS) of *CsDIR16* gene (gene ID Csa4M280630.1), and Primer Premier 5.0 (PREMIER Biosoft International, CA, USA) was used to design the specific primers for cloning the full-length CDS. The full-length *CsDIR16* ORF was amplified by PCR using the primers *CsDIR*-F (5′-ATGGCTGGAATCTCTCCAAT-3′) and *CsDIR*-R (5′-TCAATAATGAAGCACGTAAATGTTA-3′). PCR reaction was performed using the following thermocycling conditions: 94 °C for 5 min; followed by 31 cycles with 94 °C for 30s, 55 °C for 30s, 72 °C for 30s; and then 72 °C for 10 min. The amplicons were cloned into the pEASY-T3 vector (TransGen Biotech) and sequenced by GENEWIZ.

The deduced CsDIR16 protein sequence was analyzed using the Conserved Domain Database (CDD) of NCBI (https://www.ncbi.nlm.nih.gov/Structure/cdd/wrpsb.cgi). DNAMAN software (http://www.lynnon.com/) was utilized to peform sequence alignments. A phylogenetic tree was constructed by MEGA7.0 software using neighbor-joining algorithm. All sequences data were obtained from NCBI (Additional file 2: Table S4).

### Promoter sequence analysis

The promoter sequence, which was located 1410 bp upstream of the transcription start site, was obtained by a BLAST search of the cucumber genome database (http://www.icugi.org/). The online tool Plant CARE (http://bioinformatics.psb.ugent.be/webtools/plantcare/html/) was used to analysis.

### Subcellular-localization analysis

A *CsDIR16-GFP* (green fluorescent protein) vector was constructed by cloning the *CsDIR16* ORF into a pGII-eGFP vector using the primers 5′-AACGGATCCATGGCTGGAATCTCTCCAAT-3′ (HindIII site underlined) and 5′-TCCCCCGGGAATAATGAAGCACGTAAATGTTA-3′ (SmaI site underlined). The plasmids pGII-eGFP and pGII:CsDIR16-eGFP were transformed into *Arabidopsis* protoplast cells [24]. Subcellular localization in protoplasts was observed using a TCS SP2 confocal spectral microscope imaging system (Leica, Germany).

### qRT-PCR analysis

Total RNA was extracted and subjected to reverse transcription as described above. qRT-PCR was performed using SYBR® Green Realtime PCR Master Mix (Toyobo, Japan) in an iQ5 (Bio-Rad) thermocycler. The amplification conditions were as follows: denaturation at 95 °C for 10 min, followed by 40 cycles of 95 °C for 15 s and 55 °C for 15 s. Relative quantitation of gene expression was performed using *CsEF1α* (GenBank Accession Number: XM_004138916) as control [43]. Four replicates were used for each treatment. Melting-curve analysis was performed after the amplification was complete. The 2^-ΔΔCT^ method was used for analyzing the real-time qPCR results.

The following gene-specific primers were used: *CsDIR*-qF (5′-ATAGCCGAAGATGAAAACTCCT-3′) and *CsDIR*-qR (5′-TTGGACCGCACCGAATC-3′); *EF1α*-qF (5′-CCAAGGCAAGGTACGATGAAA-3′) and *EF1α*-qR (5′-AGAGATGGGAACGAAGGGGAT-3′).

### Expression vector construction and transformation of cucumbers

The plant expression vector pCXSN was used for TA cloning. The T-DNA region selection markers for hygromycin resistance were replaced by the herbicide-resistance gene *bar* [49, 50]. There were two *Xcm*I restriction sites downstream of CaMV35S promoter in pCXSN. The over-expression vector was constructed by the RT-PCR products of *CsDIR16* ligating into the pCXSN vector, which had been digested with *Xcm*I (Additional file 1: Figure S4). An overexpression vector, *CsDIR16*(+)-PCXSN, was constructed through TA cloning, as well as an antisense expression vector, *CsDIR16*(−)-PCXSN. The directionality of the target gene within the vector was confirmed by sequencing using the primers pCXSN-F (5′-CGGCAACAGGATTCAATCTTA-3′) and pCXSN-R (5′-CAAGCATTCTACTTCTATTGCAGC-3′).

The recombinant plasmids *CsDIR16*(+)-PCXSN and *CsDIR16*(−)-PCXSN were separately introduced into *Agrobacterium tumefaciens* strain LBA4404, then simultaneously transferred into ‘D9320’ cucumber cotyledons using the cucumber genetic-transformation system [48, 51], and tested for resistance to glufosinate (1 mg/L). PCR and qRT-PCR analyses were performed on the transgenic plants.

### Measurements of PM residues, POD activity, and MDA content

The level of PM residue was measured as described by Meng et al. [24]. Briefly, approximately 5.0 g of cucumber tissue was added to 25 mL of acetonitrile and homogenized with a high-speed homogenizer (Heidolph Silent Crusher-M®) for 2–3 min at 14–15000×*g*, and stood at room temperature for 1 h. 3 g NaCl was added into each extraction, then vortexed vigorously for 1 min and centrifuged for 10 min at 5000×*g*. 5 mL of each supernatant was dried with Termovap sample concentrator. 1 mL methyl alcohol was added to the residues, and then filtered through a 0.22-μm polypropylene filter. Agilent 7890A gas chromatography system (Agilent Technologies) equipped with a capillary column (HP-5, 30 m × 0.25 mm × 0.25 μm) was applied to analyze the level of PM residue. The column temperature was sustained 40 °C for 2 min, and then raised to 200 °C at the speed of 25 °C·min^− 1^, and held at that temperature for 8 min. Nitrogen was used as the carrier gas, with a hydrogen flow rate of 60 ml·min^− 1^, an air flow rate of 400 ml·min^− 1^, and a tail wind flow rate of 60 ml N_2_·min^− 1^. The injection port temperature and detection temperature were both set at 240 °C.

To determine the activities of POD enzymes, fresh leaf material (500 mg) was homogenized in 5 ml of 50 mmol phosphate buffer (pH = 7.0) containing 1% soluble polyvinylpyrrolidone. The homogenates were centrifuged at 15000×*g* for 10 min, and the POD activities in the supernatants were determined spectrophotometrically by measuring the absorbance at 470 nm, as described previously [52]. The reaction mixture contained 5 × 10^− 3^ M guaiacol and 5 × 10^− 3^ M H_2_O_2_ in 0.1 M phosphate buffer (pH = 6.0). The reaction was initiated by adding 20 μL of protein extract to 3 mL of reaction mixture. Changes in absorbance, due to the catalytic conversion of pyrogallol to purpurogallin, were measured at 30-s intervals for 3 min at 470 nm.

MDA levels were measured as described by Wu et al. [53]. The thiobarbituric acid (TBA) method was used to detect the amount of MDA. Briefly, approximately 0.5 g of sample was homogenized with 5 mL 10% trichloroacetic acid (TCA). The homogenate was centrifuged at 11,000×*g* for 15 min. 1 mL supernatant was mixed with 2 mL 10% TCA containing 0.67% TBA. The mixture was heated in boiling water bath for 15 min, then cooled immediately in an ice bath, and centrifuged at 4000×*g* for 20 min. The absorbance values of the supernatant at 600, 532, and 450 nm were determined with ultraviolet–visible spectrophotometer (Shimadzu, Japan). The quantity of MDA was calculated using the following equations:

$$ {\mathrm{c}}_{\mathrm{MDA}}\left(\upmu \mathrm{mol}/\mathrm{L}\right)=6.45\times \left({\mathrm{A}}_{532}-{\mathrm{A}}_{600}\right)-0.56{\mathrm{A}}_{450} $$

$$ \mathrm{MDA}\ \mathrm{content}\;\left(\upmu \mathrm{mol}/\mathrm{g}\;\mathrm{FW}\right)={\mathrm{c}}_{\mathrm{MDA}}\times \mathrm{V}/\mathrm{W}\;\left(\mathrm{FW}\right) $$

### Statistical analysis

All data measurements were replicated at least 3 times. The data were subjected to statistical analyses using the Origin8.0, DPS7.05 (Data Processing System), and GraphPad Prism 6 programs. Data were expressed as the mean ± SD. Significant differences between the treatment and control groups were confirmed by Student’s *t*-tests. The data were analyzed by analysis of variance (ANOVA; *p* < 0.001), followed by Tukey’s test to compare differences between the groups at a significance level of *p* < 0.05.

## Additional files

Additional file 1: Figure S1. Amino acid sequence alignment of the DIR family from cucumber, *Arabidopsis thaliana* (At) and *Cucumis melo* (Cm). Alignment generated using ClustalW (blosum matrix, gap open and gap extension penalties of 5 and 1.0, respectively) and Boxshade. Conserved similarity shading is based on 50% identity (black) and 50% similarity (gray). **Figure S2.** Signal peptide prediction of *CsDIR16* coding protein **Figure S3.** Transmembrane analysis of *CsDIR16* coding protein, **Figure S4.** Construction of plant vector *CsDIR*16-pCXSN(±), **Table S1.** Locations and sequences of cis-elements in the promoter regions of the *CsDIR16* genes, **Table S2.** Identified *CsDIR* genes in cucumber genome, **Table S3.** *CsDIRs* gene that responds to PM stress. (DOCX 433 kb)

Additional file 2: Table S4. All sequences data in Fig. 2. (DOCX 26 kb)

## Abbreviations

ABA: abscisic acid
cDNA: complementrary DNA
CDS: coding sequences
Cor: *Corynespora cassiicola* Wei
DIR: dirigent protein
GFP: green fluorescent protein
JA: jasmonic acid
PCR: polymerase chain reaction
PEG4000: Polyethylene glycol 4000
PM: propyl-[3-(dimethylamino) propyl]carbamate
qRT-PCR: real-time quantitative reverse transcription-polymerase chain reaction
RNA: ribonuncleic acid
RT-PCR: reverse transcription-polymerase chain reaction
SA: salicylic acid
Tag-Seq: high-throughput tag-sequencing
