# Supplementary material for: Expression and functional analysis of the Propamocarb-related gene CsDIR16 in cucumbers
Source: BMC Plant Biol. 2018 Jan 18;18:16. doi: 10.1186/s12870-018-1236-2 (PMC5774166; doi:10.1186/s12870-018-1236-2)
Supplement: Supplementary file 1 — Amino acid sequence alignment of the DIR family from cucumber, Arabidopsis thaliana (At) and Cucumis melo (Cm). Alignment generated using ClustalW (blosum matrix, gap open and gap extension penalties of 5 and 1.0, respectively) and Boxshade. Conserved similarity shading is based on 50% identity (black) and 50% similarity (gray). Figure S2. Signal peptide prediction of CsDIR16 coding protein Figure S3. Transmembrane analysis of CsDIR16 coding protein, Figure S4. Construction of plant vector CsDIR16-pCXSN(±), Table S1. Locations and sequences of cis-elements in the promoter regions of the CsDIR16 genes, Table S2. Identified CsDIR genes in cucumber genome, Table S3. CsDIRs gene that responds to PM stress. (DOCX 433 kb) [file 12870_2018_1236_MOESM1_ESM.docx]

**Supplementary Material**


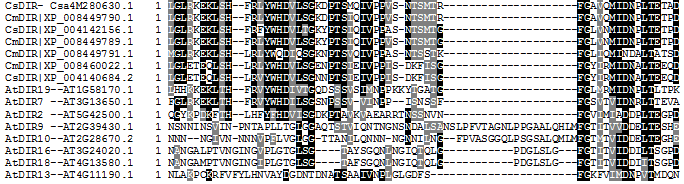

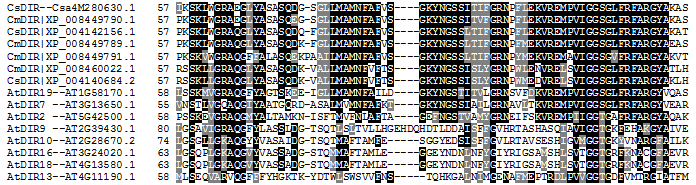

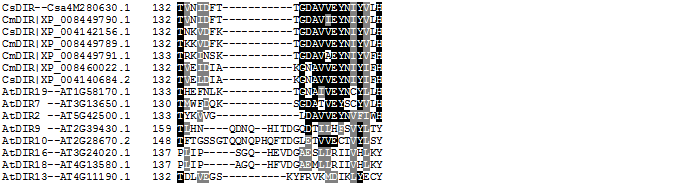


I

II

III

V

IV

**Fig. S1** Amino acid sequence alignment of the DIR family from cucumber, Arabidopsis thaliana (At) and Cucumis melo (Cm). Alignment generated using ClustalW (blosum matrix, gap open and gap extension penalties of 5 and 1.0, respectively) and Boxshade. Conserved similarity shading is based on 50% identity (black) and 50% similarity (gray).


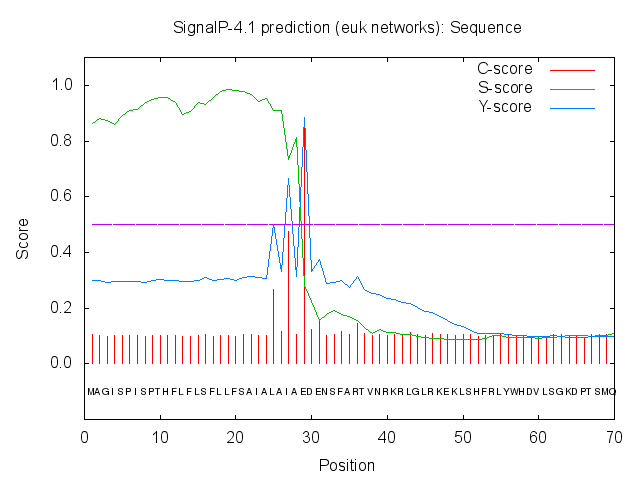


**Fig. S2** Signal peptide prediction of *CsDIR16* coding protein


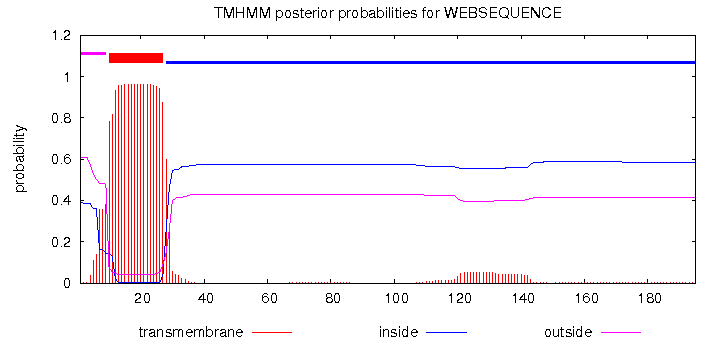


**Fig. S3**  Transmembrane analysis of *CsDIR16* coding protein


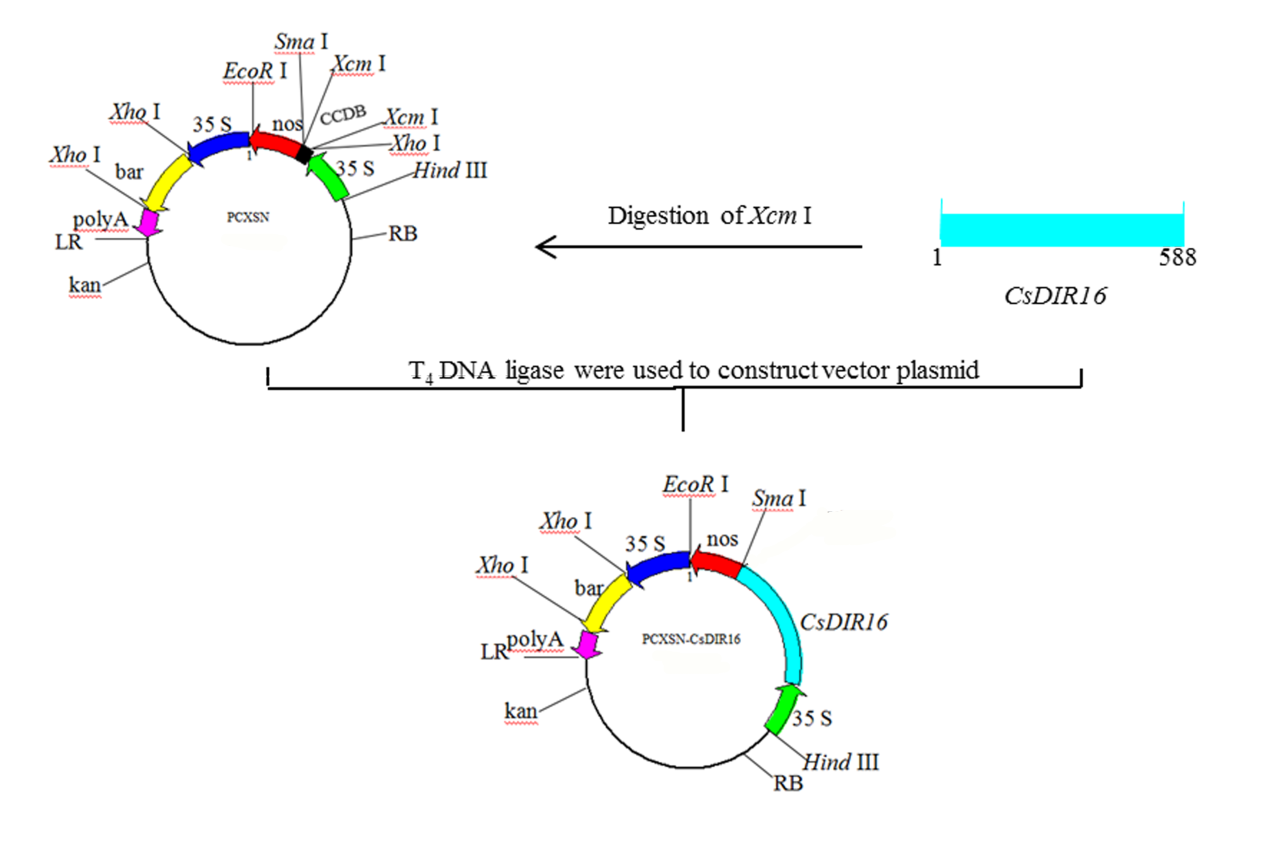


**Fig. S4** Construction of plant vector *CsDIR*16-pCXSN(±)

**Table S1** Part of cis-acting element analysis of the promoter sequences in *CsDIR16*

| component name | sequence | +/-chain | position | function |
| --- | --- | --- | --- | --- |
| Box 4 | ATTAAT | + | 572、658 | part of a conserved DNA module involved in light responsiveness |
|  |  | - | 799、1037 |  |
| Box I | TTTCAAA | + | 65、293 | light responsive element |
|  |  | - | 278 |  |
| CATT-motif | GCATTC | + | 1230 | part of a light responsive element |
|  |  | - | 997 |  |
| Box-W1 | TTGACC | + | 859 | fungal elicitor responsive element |
| P-box | CCTTTTG | - | 395 | gibberellin-responsive element |
| TC-rich repeats | G(A)TTTTCTTA(C)C(A) | - | 124、670、579 | involved defense and stress responsiveness |
| W-box | TTGACC | + | 859 | WRKY binding site |
| TCA-element | CCATCTTTTT | - | 1003 | salicylic acid responsiveness |
| TGA-element | AACGAC | - | 1091 | auxin-responsive element |

**Table S2** Identified *CsDIR* genes in cucumber genome

| Gene | Annotation ID | Gene | Annotation ID | Gene | Annotation ID |
| --- | --- | --- | --- | --- | --- |
| *CsDIR1* | Csa1M015640.1 | *CsDIR9* | Csa3M166330.1 | *CsDIR17* | Csa4M280640.1 |
| *CsDIR2* | Csa1M015830.1 | *CsDIR10* | Csa3M798110.1 | *CsDIR18* | Csa4M280650.1 |
| *CsDIR3* | Csa1M058130.1 | *CsDIR11* | Csa4M050220.1 | *CsDIR19* | Csa5M179740.1 |
| *CsDIR4* | Csa2M264050.1 | *CsDIR12* | Csa4M050240.1 | *CsDIR20* | Csa5M179750.1 |
| *CsDIR5* | Csa2M264060.1 | *CsDIR13* | Csa4M050250.1 | *CsDIR21* | Csa6M084580.1 |
| *CsDIR6* | Csa2M416780.1 | *CsDIR14* | Csa4M280610.1 | *CsDIR22* | Csa7M043060.1 |
| *CsDIR7* | Csa3M001750.1 | *CsDIR15* | Csa4M280620.1 | *CsDIR23* | Csa7M238990.1 |
| *CsDIR8* | Csa3M133310.1 | *CsDIR16* | Csa4M280630.1 |  |  |

**Table S3** *CsDIRs* gene that responds to PM stress

| Gene | Control_norm  (TPM) | PM_norm  (TPM) | LOG2 | Blast annotation |
| --- | --- | --- | --- | --- |
| *CsDIR5* | 0.05 | 1.13 | 4.50 | disease resistance response protein |
| *CsDIR7* | 2.48 | 2.26 | -0.134 | Dirigent-like protein |
| *CsDIR10* | 4.14 | 4.23 | 0.0313 | Dirigent protein 1-related |
| *CsDIR16* | 0.83 | 19.45 | 4.55 | Dirigent protein 19 |
