# Supplementary material for: Expression and functional analysis of the Propamocarb-related gene CsDIR16 in cucumbers
Source: BMC Plant Biol. 2018 Jan 18;18:16. doi: 10.1186/s12870-018-1236-2 (PMC5774166; doi:10.1186/s12870-018-1236-2)
Supplement: Supplementary file 2 — All sequences data in Fig. 2. (DOCX 26 kb) [file 12870_2018_1236_MOESM2_ESM.docx]

**Table S4.** All sequences data in Figure 2.

| **GeneBank**  **accession number** | **Protein name** | **Sequence** |
| --- | --- | --- |
| [NP_199065.1](https://www.ncbi.nlm.nih.gov/protein/15238980?report=genbank&log$=prottop&blast_rank=1&RID=0FCZMYAN015) | AtDIR2 | MAKRFLLLLPLLSTILLLSVSVTESEAYSTTKPCQGYKPDKFTHLHFYFHDVISGDKPTAVKVAEARRTNSSNVNFGVIMIADDPLTEGPDPSSKEVGRAQGMYALTAMKNISFTMVFNLAFTAGEFNGSTVAMYGRNEIFSKVREMPIIGGTGAFRFARGYAQAKTYKVVGLDAVVEYNVFIWH |
| [NP_187974.1](https://www.ncbi.nlm.nih.gov/protein/15231302?report=genbank&log$=prottop&blast_rank=1&RID=0FCV8BDY01N) | AtDIR7 | MAKLILIIVTQILLIAAVVSARKGENFAKTIDKKHFGLRKEKLTHFRVYWHDILSGSNPSSVVINPPISNSSFFGSVTVIDNRLTTEVAVNSTLVGQAQGIYAATGQRDASALMVMNFAFKTGKYNGSSIAILGRNAVLTKVREMPVIGGSGLFRFARGYVEARTMWFDQKSGDATVEYSCYVLHY |
| [NP_181475.1](https://www.ncbi.nlm.nih.gov/protein/15225466?report=genbank&log$=prottop&blast_rank=1&RID=0FD2JJV101N) | AtDIR9 | MAKALHITIFLFLISSNLLAFINSARLLDEIQPQPQLVPTGQIPTVAPTEAEEEDGTDDNPGLATTTTTASAVTVPAGPAEATEPLLEFFMHDVLGGSHPSARVVTGIVAQTEVNGIPFSKASNSIFPVDNGVPLVNSNNINSVINPNTAPLLTGLGGAQTSTVIQNTNGNSNDALSANSLPFVTAGNLPPGAALQHLMFGTITVVDDELTESHELGSAVIGRAQGFYLASSLDGTSQTLSLTVLLHGEHDQHDTLDDAISFFGVHRTASHASQIAVIGGTGKFEHAKGYAIVETLHNQDNQHITDGQDTILHFSVYLTYKA |
| [Q9SIA8.1](https://www.ncbi.nlm.nih.gov/protein/75206072?report=genbank&log$=prottop&blast_rank=1&RID=0FD5D00M01N) | AtDIR10 | MHDILGGSNPTARAVTGVVANPALSGQLPFAKPNGANLPVSNGVPSNNNNNGIVNNNNVPFLVGLGGTTANILQNNNNGNNILNGFPVASGGQLPSGSALQMLMFGTMTVIDDELTEGHELGSGLLGKAQGYYVASAIDGTSQTMAFTAMFESGGYEDSISFFGVLRTAVSESHIGVMGGTGKYVNARGFAILKTFTGSSGTQQNQPHQFTDGLETVVECTVYLSY |
| [NP_192858.1](https://www.ncbi.nlm.nih.gov/protein/15217886?report=genbank&log$=prottop&blast_rank=1&RID=0FCJZVS201N) | AtDIR13 | MANQIYIISLIFLSVLLYQSTTVLSFRQPFNLAKPCKRFVFYLHNVAYDGDNTDNATSAAIVNPLGLGDFSFGKFVIMDNPVTMDQNMLSEQVARVQGFFFYHGKTKYDTWLSWSVVFNSTQHKGALNIMGENAFMEPTRDLPVVGGTGDFVMTRGIATFMTDLVEGSKYFRVKMDIKLYECYY |
| [NP_189044.1](https://www.ncbi.nlm.nih.gov/protein/15229564?report=genbank&log$=prottop&blast_rank=1&RID=0FDA7U0U01N) | AtDIR16 | MMIKQSPFLLLTTILFTVAVFVAALDPAPEDPIFELYMHDLLGGSSPTARPITGLLGNIYNGQVPFAKQIGFTPPENGIAIPNANGALPTVNGINGVPLGTGLSGTAYSGQNLNGIQTQLGPDGLSLGFGTITVIDDILTSGPDLGSQPLGKAQGVYVASSADGSTQMMAFTAMLEGGEYNDNLNFYGIYRIGSAMSHLSVTGGTGRFKNACGFAEVRPLIPSGQHEVDGAESLLRIIVHLKY |
| [NP_193094.1](https://www.ncbi.nlm.nih.gov/protein/15236315?report=genbank&log$=prottop&blast_rank=1&RID=0FDDG9G1015) | AtDIR18 | MMKQSPFSLLTSIFLIAALFTATTALDPAPEDPIFELYMHDILGGSSPTARPITGLLGNIYNGQVPFAKQIGFVPPQNGVAIPNANGAMPTVNGINGIPLGTGLSGTAFSGQNLNGIQTQLGPDGLSLGFGTITVIDDIITSGPDLGSQPLGKAQGVYVASSADGSTQMMAFTAMLEGGEYNDNLNFYGIYRIGSAMSHLSVTGGTGRFKNACGFAEVRPLIPAGQHFVDGAEMLLRIIVHLKY |
| [NP_176113.1](https://www.ncbi.nlm.nih.gov/protein/15217886?report=genbank&log$=prottop&blast_rank=1&RID=0FCJZVS201N) | AtDIR19 | MGSFLSFFLISSRTLALVLISVTGETLESNFLHHKKEKLTHFRVYWHDIVTGQDSSSVSIMNPPKKYTGATGFGLMRMIDNPLTLTPKLSSKMVGRAQGFYAGTSKEEIGLLMAMNFAILDGKYNGSTITVLGRNSVFDKVREMPVIGGSGLFRFARGYVQASTHEFNLKTGNAIVEYNCYLLHY |
| XP_008449790.1 | CmDIR7 | MAGISPISTTHFLFLSFLLFSAVALAIAEDENSFARTVKRKRLGLRKEKLSHFRLYWHDVLSGKDPTSIQIVPPVSNTSMTRFGAVQMIDNPLTETPDPKSKLWGRAEGLYASASQEGSGLLMAMNFAFVSGKYNGSSITVFGRNPFLEKVREMPVIGGSGLFRFARGYAKASTVNIDFTTGDAVIEYNIYVLHY |
| XP_008449789.1 | CmDIR20 | MAGISPISATHFLFLSFLLSSVVALAIAEDEPSFARTVERKRLGLRKEKLSHFRLYWHDVLSGKYPTSIQIVPPVSNTSMTGFGLVNMIDNPLTETPDPKSKLWGRAQGLYASASQDKFGLLMAMNFAFVSGKYNGSSLTIFGRNPFMEKVREMPVIGGSGLFRFARGYAEASTKKVDFKTGDAVVEYNIYVLHY |
| [BAS72036.1](https://www.ncbi.nlm.nih.gov/protein/937896056?report=genbank&log$=prottop&blast_rank=1&RID=0FDGS8G801N) | OsDIR02 | MHNELYMHLYINQTISGPNPNQLVVVNGSQQAPLFFGLTAISDWTILDGPGPNASVVGRAQGMHFQSGHIREKWYTSMNFLFEDTRFNGSMLQVMGTTPQDDQWAILGGTGEFVAAEGIVEHKIVQVDCTGRIYEIKIHAFYIPMNSSAILGKKYKIW |
| [KGN63770.1](https://www.ncbi.nlm.nih.gov/protein/700208674?report=genbank&log$=prottop&blast_rank=1&RID=0FEEC3NR014)  (Csa1M015640.1 is ID in the cucumber genome database) | CsDIR1 | AMAVSSKILVISMAILVAVCSTEALDPNVTRIQFYMHDIVSGPNPTAIQVAGRQTNYAGTDPIAAMFGSVFMMDNPLTVTPELNSTLIGRAQGIYAMSAQQNEFSLLMTLTFGMTGGQYNGSSFSVVGRNPIMNEVREMPVVGGTGIFRLVRGYCLARTFSFRNMDAVIGYNVTLIHDLYDYSR |
| [XP_004138340.1](https://www.ncbi.nlm.nih.gov/protein/449441137?report=genbank&log$=prottop&blast_rank=1&RID=0FE0H472014)  (Csa1M015830.1 is ID in the cucumber genome database) | CsDIR2 | MATKTMTINLCLILLSFGVSSTTATKSYARNIDPKSLKLNNKQHQKLTHLRLYWHDTVSGGRPSSVAVLPPLNNVTEFGQVNMFDNPLTAGPELGSQLVGRSQGFYAGAAQDQIGLLMAMNFAFTHGKYKGSSLTVIGRNHISDAVREMPVVGGSGKFRFGSGYALAKTHCLDPVTFDAVVEYNVYVLHY |
| [KGN64478.1](https://www.ncbi.nlm.nih.gov/protein/700209382?report=genbank&log$=prottop&blast_rank=1&RID=0FH5D3X4015)  (Csa1M058130.1 is ID in the cucumber genome database) | CsDIR3 | MANPTNPKFPLSLFFILTAALSSADSSTTIHNQVAPNPIDQFETNLSFFMHNILGGSHPTARTVTGTIPNKAEPTAGLPFSKPKKTIFPLPGAVPLIAANRNSNEGGIANHNNKNNQPFVTAGQVPSAAVLQHVMFGSITTIDDELTEGEELGSGVMGRGQGFYFISSLDGSSHTVALTVILHRYDENEEKKDEDTISFFGVHRRGSMESPIAVVGGTGKYENASGFAVIENLRRRENQYMTDGDDTIVHFRVYLSQ |
| [XP_004147025.1](https://www.ncbi.nlm.nih.gov/protein/449458580?report=genbank&log$=prottop&blast_rank=1&RID=0FEBGW2Z01N)  (Csa2M264050.1 is ID in the cucumber genome database) | CsDIR4 | MEITKLAMGVLVLFLSAISARSEYYSKTVPRVQLKEKVTNLHFFLFDILSGKKPSAVEVAHANITIGEQSATPFGSVYAVDDPLREGPDPESKVIGNARGLYVSASQGADLCLAMYIDYGFTTGPFNGSSISVFSRNPVTEQRREVAVVGGRGKFKMARGFAKLKTHYLNVSNGDAIIEYNVTVFHY |
| [XP_004147026.1](https://www.ncbi.nlm.nih.gov/protein/449458582?report=genbank&log$=prottop&blast_rank=1&RID=0FE7YHVK015)  (Csa2M264060.1 is ID in the cucumber genome database) | CsDIR5 | MANLSVSFSFILLIATLPWIQSLNPKKPVISRHVSQKQTVTNIQFYFHDTVSGKTPSAIKVAEAPTSSKSPTLFGALFIADDPLTESPDPKSKEVGRAQGLYGSAGQQELGLLMALTYEFTAGKFKGSSVVVLGKNSVMHTVRELPIVGGTGVFRFARGYAEARTYWLNSVGDAIVGYNVTVIH |
| [XP_011649995.1](https://www.ncbi.nlm.nih.gov/protein/778673457?report=genbank&log$=prottop&blast_rank=1&RID=0FEK5175014)  (Csa2M416780.1 is ID in the cucumber genome database) | CsDIR6 | MDTNFTTKLSLALILTAAAVSDTTARKSKHTNLILYVQDFANGPNPTFIPVAGVAGKPWNFTQFGTIFVTDNPITAGPDRNSRALGRAQGMYVVAAADGRNLAVILTLALAEGSSIEIQGTSRQFEGVRELGVVSGTGKFRFVRGFAVGKNVVTDIANGYTVVQFNVSLKHY |
| [KGN55603.1](https://www.ncbi.nlm.nih.gov/protein/700200470?report=genbank&log$=prottop&blast_rank=1&RID=0FGVPDV8015)  (Csa3M001750.1 is ID in the cucumber genome database) | CsDIR7 | MEREPVWCLKRKVILAMLMLCKPSKLEVMEVPSDVPTRFEARSAPVVKGSWKAKMVPNGVVVWLTPGPTTPGTMK |
| [XP_004134021.1](https://www.ncbi.nlm.nih.gov/protein/449432468?report=genbank&log$=prottop&blast_rank=1&RID=0FGY225E015)  (Csa3M133310.1 is ID in the cucumber genome database) | CsDIR8 | MRQASFPAFLATAIIVVHFVAFRSAAVTANVGAHEPVLEFYMHDILGGASPTARPITGLLGNIYTGQVPFATPIGFLPPDGGVAIPNANGALPTVNGINGIPLGTGLSGTSFAGNPNPQNVPQTQLGPDGLGLGFGTITVIDDVLTTSPELGSQSIGQAQGVYVASSADGTTQMMAFTAMVEGGEYGDSLNFYGVFRIGSPSSHLSVTGGTGKFKNARGIAEVRSLIPPGQHVADGAETLLRVLVHLTY |
| [XP_004134245.1](https://www.ncbi.nlm.nih.gov/protein/449432918?report=genbank&log$=prottop&blast_rank=1&RID=0FF7W6WM01N)  (Csa3M166330.1 is ID in the cucumber genome database) | CsDIR9 | MAVSDRLTFTNFLLLSIIALYFSNGFPNLFNPQPNQTNLVVYVHDYFTGEDASAITVGGRKGPESSVLEFGTQMVVDDLVTEGPKIDSREIGRAQGMYINSQSDGKGLYMVFSVIFSGGEFRGSSLEIQGPDLFTMKEREFGVVSGTGFFRFVKGFGIMQTESMDLVHLRAVIKLNITVNHY |
| [XP_004153955.1](https://www.ncbi.nlm.nih.gov/protein/449473693?report=genbank&log$=prottop&blast_rank=1&RID=0FDX066B01N)  (Csa3M798110.1 is ID in the cucumber genome database) | CsDIR10 | MAPILIPMPSTAVAAATFFSLFLTSLLAASDTVLSPEKLGIRRREKLSHLHFYFHDIVSGRNPTALIVVPPPSSNASRTLFGAVVMTDDPLTERPEIGSKLLGKAQGFYAGASKTEFGLLMVMNFAFVEGKYNGSYLSILGRNTIMSAVREMPVVGGGGLFRFARGYALAKTHALNFSSGDAVVEYNVYVFHY |
| [XP_004146539.1](https://www.ncbi.nlm.nih.gov/protein/449457606?report=genbank&log$=prottop&blast_rank=1&RID=0FFRDRYB015)  (Csa4M050220.1 is ID in the cucumber genome database) | CsDIR11 | MSLKSPISNLFFFVFLLLITFSVASKHRFRRPCRHLVFYFHDIIFNGHNAKNATSAIVGAPAWGNLTVLAAQNHFGNVVVFDDPITLDNNLHSPPVGRAQGFYIYDKKDIFTAWLGFSFVFNSTEHRGSLNFAGADPLMNKTRDISVIGGTGDFFMARGIATLSTDSLEGEVYFRLRTDIKLYECW |
| [XP_004146540.1](https://www.ncbi.nlm.nih.gov/protein/449457608?report=genbank&log$=prottop&blast_rank=1&RID=0FG0P2JP014)  (Csa4M050240.1 is ID in the cucumber genome database) | CsDIR12 | MAFSTKNCITITVLALFLFSSSSCSALPMVKKQKHKPCKQLVLYFHDVLYNGKNAKNATSAIVAAPEGANLTILAPQFRFGNIVVFDDPITLDNNLHSNPVGRAQGMYIYDTKNTFTVWLAFSFSLNYTAYKGTINFVGADPILVKTRDISVVGGTGDFFMHRGVATIMTDAFEGEVYFRLRVDIKFYECW |
| [XP_004146540.1](https://www.ncbi.nlm.nih.gov/protein/449457608?report=genbank&log$=prottop&blast_rank=1&RID=0FFUV75V014)  (Csa4M050250.1 is ID in the cucumber genome database) | CsDIR13 | MAFSTKNCITITVLALFLFSSSSCSALPMVKKQKHKPCKQLVLYFHDVLYNGKNAKNATSAIVAAPEGANLTILAPQFRFGNIVVFDDPITLDNNLHSNPVGRAQGMYIYDTKNTFTVWLAFSFSLNYTAYKGTINFVGADPILVKTRDISVVGGTGDFFMHRGVATIMTDAFEGEVYFRLRVDIKFYECW |
| [KGN54084.1](https://www.ncbi.nlm.nih.gov/protein/700198926?report=genbank&log$=prottop&blast_rank=1&RID=0FENVUS701N)  (Csa4M280610.1 is ID in the cucumber genome database) | CsDIR14 | MAPVSNTSRTRFGAVQMIDNPLTETQDLPKSKLWGRAQDQSGLLMAMNLAFVSGKYNGSSVTIFGRNPFMEKVRERCL |
| [KGN54085.1](https://www.ncbi.nlm.nih.gov/protein/700198927?report=genbank&log$=prottop&blast_rank=1&RID=0FDUGR6A014)  (Csa4M280620.1 is ID in the cucumber genome database) | CsDIR15 | MISISMAIISPISATHFLFLSFLLSFAIAEHEHSFARTMDRKLLGLEKEKLSHLRLYWHDVVSGKNPTSVQIVAPVSNTSRTRFGAVQMIDNPLTETKDPKSRLWGRGQGFYASASQDKLGLLMAMNLAFVSGIQRKLNRYIWKKPSWGESERDVCDRWRWSFQICERVCKSHNQEVQFQNRRCCC |
| [XP_004142157.2](https://www.ncbi.nlm.nih.gov/protein/778693009?report=genbank&log$=prottop&blast_rank=1&RID=0CK67G66014) (Csa4M280630.1 is ID in the cucumber genome database) | CsDIR16 | MAGISPISPTHFLFLSFLLFSAIALAIAEDENSFARTVNRKRLGLRKEKLSHFRLYWHDVLSGKDPTSMQIVPPVSNTSMTRFGAVQMIDNPLTETADIKSKLWGRAEGLYASASQDGSGLLMAMNFAFVSGKYNGSSITIFGRNPFLEKVREMPVIGGSGLFRFARGYAKASTVNIDFTTGDAVVEYNIYVLHY |
| [XP_004142156.1](https://www.ncbi.nlm.nih.gov/protein/449448806?report=genbank&log$=prottop&blast_rank=1&RID=0CKXM0R8015)  (Csa4M280640.1 is ID in the cucumber genome database) | CsDIR17 | MAGISPISTTHFLFLSFLLFSAVALAVAEDQNSFARTVNRKRLGLRKEKLSHFRFYWHDVLTGKYPTSIQIVPPASNTSMTGFGLVNMIDNPLTETPDPKSKLWGRAQGLYASASQDQFGLLMAMNFAFVSGKYNGSSLTIFGRNPFLEKVREMPVIGGSGLFRFARGYAKATTNKVDFKTGDAVVEYNIYVLHY |
| [XP_011653564.1](https://www.ncbi.nlm.nih.gov/protein/778693014?report=genbank&log$=prottop&blast_rank=1&RID=0FE2Z78Y01N)  (Csa4M280650.1 is ID in the cucumber genome database) | CsDIR18 | MGSKFFFTCCLLWCLISSAMASDDDSFATRLNPKVLKLKKEKLTRFHLYWHDVVGGSNPTSVPVLPRLNNVTLFGLINMFDNPLTVGPDPKSRLVGRSQGLYASTAQHEIGLLMAMNFAFTYGKYKGSSITILGRNPILNQVREMPVVGGTGRFRFAKGHALAKTQYFNATTLDAVVEYDIYVLHYY |
| [KGN50509.1](https://www.ncbi.nlm.nih.gov/protein/700195332?report=genbank&log$=prottop&blast_rank=1&RID=0FH88Y07014)  (Csa5M179740.1 is ID in the cucumber genome database) | CsDIR19 | MANLHYSRSLLIKIIANFLIFFTTIITLSSAARTFENPTQNRHYRHHRLSFSMRDVFNSTNHHYSPTKSTMTNKQLPFSKPLGFFPPNQGIPISQTYPTTGSFSSQTPDFSTIGISFPSRSTLQELEYGMVRGIDEELFEISKSKPHVIGRIEGFYVENSEENSGGHMMGMTMYFGKGEAKDGIRVFGVYRSDHVKESHVAIIGGFGKYHGANGYATLKKSFRTKFGGKYNKFIEFNVYLSK |
| [XP_004147710.2](https://www.ncbi.nlm.nih.gov/protein/778700918?report=genbank&log$=prottop&blast_rank=1&RID=0FGK2EF2014)  (Csa5M179750.1is ID in the cucumber genome database) | CsDIR20 | MARTSLHFFFFFLLVSFYGLRSAIAARILMDDDDADAESQPQTAAVTPPLATISSPATTFPATQGGTTTLPSITGSSIPATTPSPTATNDDEEDDSVIPQTNQPVAATNNLGQTQDDQEDDSATTTPAAVSPAAAVPTLPSPPTEPLPAAVKGPEPISFYMHDILGGSHPSARVVTGIVANSDSSGIAFSKPNDNFFPIQGTLPLLNNDNLKNIINNNNNLPFLAGFNGVAQGNNLLLQNSANNGVLNGDEDNNQPFVTAGQLPSRVTLQQLMFGSVTVVDDELTEGHELGSAVVGRAQGFYMASSLDGTSQTVALTALFHSGGHEHVVEDSISFFGVHRTAMAGSQIAVVGGTGKYENARGYATVEMLHHQEDQHTTDGMDTIIHFSVYLTEE |
| [XP_004140684.2](https://www.ncbi.nlm.nih.gov/protein/778711460?report=genbank&log$=prottop&blast_rank=1&RID=0FDKTK7M014)  (Csa6M084580.1 is ID in the cucumber genome database) | CsDIR21 | MTIKSITCFILLRRADLLRKENMTASISANHFLFLFLFLILSSSIAYTQQDEAFFINKSMDKKLLGLETEQLSHLRVYWHDVLSGNNPTSIEIVPPISDKFISGFGYIRMIDNALTEEQDRSSKLLGRAQGLYASASQDKVALLMAMNFVFTSGKYNGSSISLYGRNPWMEDVRELSVIGGSGLFRFARGYAKLHTVELDIAKGNAVVEYNIYIFHYADSIALF |
| [XP_004144592.1](https://www.ncbi.nlm.nih.gov/protein/449453696?report=genbank&log$=prottop&blast_rank=1&RID=0FFMNFE2015)  (Csa7M043060.1 is ID in the cucumber genome database) | CsDIR22 | MAQNNIPSILIFCLAASISMTLLLSAEAKRTRFTIYLQDYAFGPNTTFFPIAGLPGSTLNFTDFGTLFVTDDSITTISNEGAPEIGRAQGIYVVTDKGGKNLLVLLSFVFTGGAFNGSSIEIQGTSRQFELIRELPVIAGTGKFRLARGYIRTDNFFFDPERGYSVIQVNVTLV* |
| [XP_004139895.1](https://www.ncbi.nlm.nih.gov/protein/449444264?report=genbank&log$=prottop&blast_rank=1&RID=0FHBWHXH014)  (Csa7M238990.1 is ID in the cucumber genome database) | CsDIR23 | MQPVTIPDSGVFVFRRMLTKGPENTSQIVGNAQGFIIPSEQFARSSFNIIYLSFNTPEYSGSLGVHAKHIGHENREEMTVVGGTGSFAFAQGVAIFLQTERQTFNSDTSYHLKLQLQFPK |
